# Aducanumab, Accelerated Approvals & the Agency: Why the FDA Needs Structural Reform

**DOI:** 10.1017/jme.2024.20

**Published:** 2023

**Authors:** Matthew Herder

**Affiliations:** 1: DALHOUSIE UNIVERSITY HALIFAX, NS, CANADA.

**Keywords:** Food And Drug Administration, Accelerated Approvals, Drug Regulation, Alzheimer’s Disease

## Abstract

The US Food and Drug Administration’s controversial decision to grant accelerated approval to aducanumab (Aduhelm), a therapy for Alzheimer’s disease, has motivated multiple policy reforms. Drawing a case series of other drugs granted accelerated approval and interviews of senior FDA officials, I argue that reform should be informed but not defined by aducanumab. Rather, structural reforms are needed to reshape FDA’s core priorities and restore the regulatory system’s commitment to scientific rigor.

The history of biopharmaceutical regulation is often told by way of example. Drugs that cause tragedy have harnessed the political will necessary to spawn legislative reform. In the 1960s thalidomide crystallized the case for sweeping changes to the *Food, Drugs & Cosmetics Act*, including a requirement that “substantial evidence” of effectiveness be provided prior to drug approval. However, undue attention to a particular drug — even one as harmful as thalidomide — can overlook the underlying institutional story that not only predates the regulatory (in)action in question, but also permeates through any reforms that the episode precipitates. Case in point: the US Food and Drug Administration (FDA) had begun to require proof of effectiveness from drug manufacturers several years prior to the 1962 legislative amendments motivated by thalidomide.^1^ Focusing on discrete examples — in isolation from the regulator’s institutional practices and the larger political economy in which they operate — thus runs the risk of either ignoring solutions that are already available to a regulator, or impoverishing those that are newly fashioned by the legislature to redress some shortfall in biopharmaceutical regulation. Absent deeper knowledge of institutional practices and the structures that drive them, reforms crafted hastily in response to a given drug may miss the mark.

The aim of this article is to supply a deeper institutional understanding of FDA as reforms are developed in the aftermath of the agency’s controversial decision to approve an Alzheimer’s drug known as aducanumab (Aduhelm) in June 2021. Initially carrying an annual price tag of $56,000 per patient, the agency’s decision to approve the drug with no clinical evidence that it effectively treats the disease has quickly translated into an investigation into how the controversial decision was made,^2^ concrete policy proposals to reform the “accelerated approval” pathway by which aducanumab entered the US market,^3^ and Congressional scrutiny for “regulatory capture” and the cost implications of Medicare spending on this expensive, unproven drug.^4^ Taking the offensive, the FDA has in turn publicly defended its decision to approve aducanumab.^5^ Revealing anew that not only are FDA’s decisions political, but that the agency is itself a political animal — with a reputation and set of institutional preferences that it works to maintain^6^ — I argue that it is critical to grasp that underlying reality in order to properly define the scope and specifics of any reforms that follow aducanumab’s approval.

The accelerated approval pathway is an appropriate starting point for that inquiry. At present, FDA may grant accelerated approval to a drug that targets a “serious or life-threatening condition” on the strength of evidence showing a positive impact on an (unvalidated) surrogate endpoint, which the agency has determined is “reasonably likely” to predict “clinical benefit.”^7^ In return, sponsors are generally required to conduct one or more post-approval studies to “confirm” the clinical benefit of the drug upon the disease in question. Representing a delicate balance between patients facing a dire prognosis and the agency’s core function of motivating sponsors to produce useful information about the safety and efficacy of new therapies, the integrity of the accelerated approval program rests on the prospect of securing additional evidence in the post-approval setting. However, reviews show that post-approval studies that are required under the pathway to confirm a drug’s efficacy regularly are often completed several years after the date of approval.^8^ When they are completed, the quality of the evidence that is generated in the postmarket setting often does not improve upon the evidence related to efficacy that already existed.^9^ Despite these clear shortcomings, limiting reforms *a priori* to the accelerated approval pathway risks underestimating both the magnitude of the problem currently arresting the agency and FDA’s capable resistance to reform that does not conform to its own institutional priorities. I argue, then, that FDA reform should be informed — but not defined — by the aducanumab decision. Rather, reform should be premised upon “empirical assessment” of the various barriers that impede timely completion of post-approval studies^10^ and critical attention to FDA — as an institution with defined interests and strategies — that is embedded within a larger political context.

I argue, then, that FDA reform should be informed — but not defined — by the aducanumab decision. Rather, reform should be premised upon “empirical assessment” of the various barriers that impede timely completion of post-approval studies and critical attention to FDA — as an institution with defined interests and strategies — that is embedded within a larger political context.

To develop this argument I draw upon a case series of other drug accelerated approval decisions that pre-date aducanumab; namely, treatments for acute myeloid leukemia (gemtuzumab ozogamicin); metastatic breast cancer (bevacizumab); and Duchenne muscular dystrophy (etiplersen). These therapies were selected due to the high amount of information that is now publicly available about what happened inside the agency at the time of each approval, as well as the significant public attention they each garnered. I also integrate into the cases relevant interview data from previous research involving 23 senior FDA officials, which examined the agency’s shift toward “lifecycle regulation,” in particular, FDA’s increasing reliance upon “postmarketing requirements,” including in the context of accelerated approvals, and postmarketing commitments, to secure additional evidence following market approval.^11^ The interview data reported below — only a minor portion of which was previously published — thus serves to bring into focus a number of the key institutional challenges and dynamics that lie behind the approval of gemtuzumab, bevacizumab, etiplersen, and aducanumab. Bridging the three cases and qualitative data together with an intermediate section characterizing FDA as both a captive and powerful actor, I turn to critically appraise the key elements of recent proposals to reform the accelerated approval pathway.

I conclude by making the argument about why more structural reforms are needed in view of the challenges that aducanumab, other accelerated approvals, and the political economy in which the agency resides, have brought to light. In particular, I argue that two structural changes promise more fundamental change. The first would seek to recalibrate the agency’s priorities through a revised approach to user fee legislation. At present, user fee legislation requires the agency to prioritize review time targets, which creates delays in, and takes resources away from, the timely design and enforcement of post-approval study requirements. I suggest that user fee legislation should be redesigned in order to reshape the agency’s workflows, in particular, by placing review timelines that normally apply to accelerated approvals on hold unless and until an agreed upon post-approval study design is in place. A second, more radical proposal would be to force FDA to cede some of its control over the regulatory process. Specifically, the agency would assign to an outside body which is independent of both FDA and industry the tasks of designing studies to confirm clinical benefit and deciding which indications that have been granted accelerated approval subsequently demonstrate sufficient clinical benefit to stay on the market. The agency has enlisted outside actors in the past, most notably, to assist in the task of deciphering which therapies — in the wake of reforms triggered by thalidomide — were effective enough to remain in clinical use. After aducanumab, and the close nexus between industry and FDA that the Alzheimer drug’s approval betrayed, a similar initiative stands to add scientific rigor to the regulatory system.

## Three Controversial Accelerated Approvals Before Aducanumab

### Gemtuzumab Ozogamicin: Undefined Post-Approval Study Protocols and Protracted Study Timelines

Thirty years of experience with the accelerated approval pathway shows that the process of designing, conducting, and completing post-approval studies is often riddled with delays. Consider the case of the monoclonal antibody therapy gemtuzumab ozogamicin (Mylotarg), which the FDA granted accelerated approval in May 2000 for the treatment of acute myeloid leukemia (AML). The approval letter specified that a randomized controlled trial was required to verify the antibody-drug conjugate’s efficacy against AML but left it up to the sponsor to determine the trial’s design. The trial did not begin until 2004 then proceeded slowly over five years, only to be stopped prior to completion when clinical benefit was not shown and a number of participants in the gemtuzumab arm of the trial died due to treatment toxicity.^12^ In June 2010 — a decade after its approval — Pfizer agreed to voluntarily withdraw gemtuzumab from the market, becoming the first, and only one of five withdrawals in the first 25 years of the accelerated approval pathway’s existence.^13^ Seven years later, after two new trials with a different dosing regimen demonstrated clinical benefit, including improvement in overall survival, FDA granted a standard (not accelerated) approval for a new, lower dose version of gemtuzumab in September 2017.

Why it took four years to initiate the study, ten years to withdraw the approval, and seventeen years to find the right dosing regimen and assess gemtuzumab’s impact on overall survival amongst AML patients is not clear. FDA has improved its practices since gemtuzumab: anticipated timelines for final protocol submission and study completion are now generally specified at the time of approval although the precise details of the post-approval study are seldom settled by that stage^14^ because FDA is reticent to slow down the approval.^15^ Recent evaluations of the accelerated approval program find that most post-approval studies are completed within 3-5 years after market entry; and in the event that withdrawal proves necessary, it is likely to occur much sooner if the post-approval study is underway by the time of approval.^16^

Notable exceptions to these improving trends exist.^17^ As well, the FDA set the date of completion for the post-approval trial to confirm aducanumab’s clinical benefit against Alzheimer’s disease in 2030 — nine years after its approval in 2021.^18^ Even when the study completion date is closer to the norm of four years, that can still represent a significant delay: Patients may be exposed to unforeseen safety risks or forego other treatment options while taking new drugs that ultimately do not serve their intended purpose. Further, the very existence of an accelerated approval may undermine ongoing trials of other products targeting the same indication, as physicians are less likely to recommend that patients should enrol in a trial when a therapy for the same condition is on the market.

The enduring delays associated with accelerated approvals point to the agency’s core priorities. There is a forward momentum that characterizes the agency’s workflow. It is, as FDA Principal Deputy Commissioner Dr. Janet Woodcock explained in an interview, a “fire drill at the end of the review process.” “FDA has worked diligently to put into place the procedures, personnel, and tracking systems in an effort to ensure postmarket studies receive adequate attention,” another official stressed, but “a breakthrough therapy [investigational new drug application] is likely to be given higher priority by officials than reviewing a postmarket study protocol.” The breakthrough therapy is “the squeaky wheel that gets the grease.”

As a result, important insights that agency reviewers have learned during the premarket review process may be lost. Focused on ensuring that the review timelines prescribed by user fee legislation are met, the agency shifts the burden of post-approval study design to the sponsor in order to allow reviewers to turn to the next priority review in the queue.^19^ It is not that FDA’s guidance on the particulars of postmarket study design is unwelcome; rather, companies do not want that guidance within the four corners of the approval letter because, as one former director noted, “that letter is posted on the FDA’s website.” Instead, sponsors prefer to work out the finer details of post-approval study design iteratively — in confidence — with the agency in the weeks and months following approval.^20^ Meanwhile, the agency has the discretion to determine on a case-by-case basis whether any delays that ensue, for instance, with respect to recruiting patients to participate in the post-approval study, are for “good cause” or merit enforcement action. To date, enforcement actions have been exceedingly rare.^21^

Following the experience with gemtuzumab, it became the agency’s goal to have post-approval study design completed and trial recruitment underway at the point of approval. FDA leadership has, in the wake of aducumab, articulated that as a firm expectation while allowing for some continuing flexibility around whether the confirmatory study needs to be “substantially” versus “fully” enrolled at the point of approval depending on whether the therapy is a breakthrough therapy or “just another drug.”^22^ Having full (or substantial) study enrollment complete by approval entrenched in law as a requirement would, however, take internal resources away from high priority reviews, and disrupt the settled division of labour as between the agency and sponsors. Absent added resources and adjusted timelines, the agency likely has little appetite for legislation stipulating that post-approval study designs should be in place by the point of approval.

### Bevacizumab: Withdrawing Accelerated Approvals in the Face of Patient Opposition

Under the law, accelerated approvals come with the power to withdraw the approved indication in an equally expeditious manner.^23^ FDA has the power to withdraw an indication when a post-approval study, required as part of the accelerated approval process, is not completed or fails to confirm the efficacy of the drug. In the thirty year history of accelerated approvals, such withdrawals have occurred on only rare occasions: According to a twenty-five year review of all hematology and oncology related accelerated approvals, only five (of 93) indications were withdrawn during that period.^24^ An additional 16 indications have been withdrawn during the last two years when accelerated approvals have been under greater scrutiny.^25^ Like gemtuzumab, all but one of these indications have been voluntarily withdrawn by the sponsor; either because new safety issues arose in the postmarket setting or for business reasons.^26^ The lone case (bevacizumab (Avastin)) of a withdrawal enforced by FDA due to a demonstrated lack of efficacy has had a lasting impact on agency thinking.^27^

Bevacizumab carries several FDA-approved indications; in 2008, metastatic breast cancer was added to the list pursuant to the FDA’s accelerated approval pathway. Bevacizumab’s sponsor Genentech carried out the postmarket study with relative efficiency. From FDA’s perspective, however, the study’s findings failed to confirm the drug’s efficacy against that form of breast cancer. Some patients and Genentech, in contrast, contended that the therapy was still beneficial. Correctly anticipating the opposition that would follow from Genentech, breast cancer patients, and others, FDA elected to hold a public hearing. The hearing proved highly controversial and patients and family members questioned the agency’s very legitimacy to interfere with the patient-doctor relationship.^28^ But the FDA Commissioner at the time held firm to the agency’s assessment of the evidence and ordered the indication’s withdrawal on the strength of a 69-page decision.^29^

To outsiders this attested to the FDA’s lasting commitment to public health.^30^ For officials on the inside, though, bevacizumab’s withdrawal is remembered as extremely labor-intensive and adversarial.^31^ Officials that I interviewed expressed different views about whether the agency was likely to pursue similar action in the future. But it was clear that the prospect of a withdrawing an accelerated approval weighed heavily on the regulator. As one official described the episode with bevacizumab:

[I]t was Armageddon…it was a worthy action and we went through with it, but it was bloody, and to me…the idea of bloodying a company who, basically their product was approved…with the understanding that once the post-market study was done, if it didn’t work as intended, product comes off the market. You agree to that…but the companies view it as a vested right. […] And for the agency to take that away, I think at times, scares the agency.

However hesitant FDA may be to follow through with a withdrawal in the future, two intertwined aspects of how the agency arrived at its bevacizumab decision are worth highlighting. The first aspect concerns the specific procedure that the agency deployed to make its decision. Known as “separation of functions,” this procedure entails creating a strict firewall between the team of scientific reviewers within the agency that made the recommendation to withdraw the indication and an independent official chosen by the FDA Commissioner to hear the dispute between the reviewers and the sponsor, Genentech.

In essence, separation of functions, which applies to some (but not all) FDA decision-making by virtue of the Administrative Procedure Act, aims to separate out the agency’s “investigative” (embodied by FDA reviewers) and more “prosecutorial” functions (vested in the Commissioner) for a defined period of time.^32^ The aim is to make the decision that is ultimately rendered as fair as possible through the adoption of various procedural safeguards that are designed to provide those affected by FDA’s decision with adequate opportunities to be heard and to prevent FDA scientific staff, who have a deep familiarity with the file, from pre-determining the Commissioner’s final decision. When separation of functions is in effect, FDA’s Office of Chief Counsel assigns select lawyers to the Center responsible for the drug in question and others to the Commissioner. Notice — both to the sponsor and the public more broadly — is provided in advance of an oral hearing, where presentations by FDA staff, the sponsor, and affected patients are heard, cross-examination of witnesses can occur, and the full rules of evidence are observed before a “presiding officer,” which can be an FDA official that was not involved in the drug’s review or an administrative law judge (ALJ) appointed by the agency. As well, any contact by FDA with outsiders about the pending regulatory action must be summarized in writing and added to the administrative record, which, together with all of the materials and transcript from the oral hearing, serves as the foundation for the Commissioner’s decision to be rendered after the hearing concludes in consultation with the presiding officer or ALJ. As a result of these procedural safeguards, separation of functions may delay regulatory action relative to other types of FDA decision-making. However, even if the decision-making timeframe is in some cases comparable to other types of FDA decisions, the demands that separation of functions places upon the regulator’s resources and operations are not (see **Table 1** for a comparison of different FDA decision-making procedures).

**Table 1:** A comparison of select administrative decision-making procedures utilized by FDA.**33**

Decision-Making Procedure	Key Procedural Elements	Examples & Corresponding Timeframes
Clinical Investigator Administrative Actions – Disqualification	•Notice to the investigator of initiation of proceedings and opportunity to explain (NIDPOE) via an informal conference, with an opportunity to resolve by consent agreement. •Notice of opportunity for an informal “Part 16” hearing; Commissioner appoints a presiding officer who can issue a summary decision or move for a hearing in the event that there is a “substantial issue of fact” at issue. •During the Part 16 hearing FDA staff as well as the investigator can present oral or written information. Reasonable and direct cross-examination is allowed, but the formal rules of evidence do not apply. •The presiding officer compiles a report and makes a recommendation to the Commissioner who makes the decision about whether to disqualify the investigator.	Following an inspection by CDER staff in 2002, FDA identified information that Dr. Lim Hendrick repeatedly and/or deliberately violated federal regulations in her capacity as an investigator in studies of the new drugs Augmentin SR and Oral Cefuroxime Axetil. A NIDPOE letter was issued on May 11, 2006 followed by a Notice of Opportunity for a Hearing (NOOH) on July 1, 2009. 164 days after the NOOH was issued, on December 11, 2009, Dr. Hendrick was disqualified as an investigator.
Accelerated Approval Withdrawal Proceedings	•Notice to the sponsor of an opportunity for a hearing on a Center’s proposal to the withdraw the approval of an application. •Sponsor must submit data and information to be relied upon at the hearing within 30 days of notice of opportunity for a hearing. Separation of functions “will not apply” at any point inf withdrawal proceedings. •Advisory committee will be present at thee hearing. •Presiding officer, advisory committee, the sponsor, or the Center may question any person who makes a presentation at the hearing. •The presiding officer compiles a report and makes a recommendation to the Commissioner who makes the decision about whether to withdraw the approval.	On February 5, 2021 FDA granted accelerated approval for umbralisib (Ukoniq) for the treatment of relapsed or refractory marginal zone lymphoma in adult patients. One year later, while confirmatory studies were ongoing, FDA issued a drug safety communication about a possible increased risk of death associated with the drug. On March 10, 2022, FDA published a notice of an advisory committee meeting to be held on April 22, 2022. In the interim, FDA met with the sponsor, who in turn submitted its request for a voluntary withdrawal on April 15, 2022, which became effective on May 5, 2022 — 83 days after the advisory committee process was initiated. In contrast, then-Commissioner Hamburg’s decision to withdraw bevacizumab was rendered on November 18, 2011, 192 days after the notice of the public hearing was published (May 11, 2011), and 339 days after FDA recommended withdrawal (December 15, 2010).
Formal Evidentiary Public Hearings	•Notice of opportunity for a formal evidentiary hearing about whether to adopt or modify a regulation, or render an order under the legislation is published, with an opportunity for persons to object to holding the hearing in question. •Commissioner is required to review all objections and determine whether the regulation should be modified or revoked, whether a hearing is justified, and whether a hearing (if requested) before a Public Board of Inquiry or an advisory committee is justified. •Hearing is to be granted if there is a “genuine and substantial issue of fact for resolution at a hearing,” which can be “resolved by available and specifically identified reliable evidence.” •If justified, Notice of the hearing must be published, including the parties to the hearing, the issue of fact on which the hearing will focus, the name of the presiding officer, and other key details about the hearing. •Persons appearing at the hearing must file a notice of participation, can be represented by legal counsel, and must declare their specific interest in the matter. •The hearing takes place in according with “Part 12” of the regulations, which specify the functions of the presiding officer, how and when parties’ submissions are to be filed, when prehearing conferences should occur, how witnesses are to give testimony under oath, what the applicable burden of proof is, what constitutes the administrative, record, how final decisions are to made or appealed, and other key procedural details.	On July 29, 1977, Donald Kennedy, FDA Commissioner at the time, issued his decision that “Laetrille” (vitamin B-17) was a “new drug” within the meaning of the Food, Drug and Cosmetic Act, and not exempt from requirements to demonstrate safety and efficacy for its intended use. FDA convened the public hearing following a court order requiring the same (Rutherford v. United States, 424 F. Supp. 105 (W.D. Okla. 1977)).Notice of the hearing was published on February 18, 1977, and at the hearing on May 2, 1977, separation of functions was followed in accordance with 21 C.F.R. 10.55. Commissioner Kennedy’s decision was published on August 5, 1977 — 170 days after the notice of the hearing was issued.

Contrary to what occurred in the case of bevacizumab, the regulations underpinning the accelerated approval pathway expressly stipulate that the separation of functions procedure “will not apply at any point in withdrawal proceedings.”^34^ The logic being that accelerated approvals should be subject to expeditious withdrawal if the promising evidence is not borne out in the post-approval trial. As one former official involved in the drafting of the original regulations recalled:

Early on in the accelerated approval regulations, not only was there going to be accelerated approval, but if the things that were required were not met […] then there was going to be accelerated withdrawal. But not with no procedure. *The process involved having, not a formal evidentiary hearing for the ALJ or whatever, but an informal hearing that would also include an advisory committee.* […] The notion of the expedited withdrawal was included in the law in section 506. The procedure itself was as described in the regulations. […] Even that expedited procedure is something that does take considerable effort, but not nearly the same amount of resources and time that the formal evidentiary hearing would have taken. (emphasis added)

Yet, contrary to that rationale and — as the official’s comments highlight — the explicit wording of the law, the FDA adhered to its separation of functions procedure in the lead up to its decision to withdraw bevacizumab’s metastatic breast cancer indication. According to then-Commissioner Dr. Margaret Hamburg, FDA chose to follow the separation of functions in order to “protect the independence of the Commissioner’s decision and make the process transparent.” This is in line with the purpose of the separation of functions procedure, but out of step with the wording of the accelerated approval regulations. Commissioner Hamburg described the decision-making process behind bevacizumab’s withdrawal as follows:

Under separation of functions, I as Commissioner (and those assisting me on this issue, such as Dr. Midthun, the Director of FDA’s Center for Biologics Evaluation and Research who served as the presiding officer at the hearing) communicated with [the Center for Drug Evaluation and Research (CDER)] about the subject of this hearing only as part of the formal hearing record, in exactly the same way that we communicated with Genentech. CDER presented its views as a party in the hearing, as did Genentech. As the applicant, Genentech was a motivated, knowledgeable, and well represented proponent of its view. Both CDER and Genentech presented evidence at the hearing and challenged each others’ presentations. In addition, members of the public submitted comments to the docket and testified at the hearing. That created the record that led to my own decision as Commissioner. I did not know, until review of that record and discussion of the issues with Dr. Midthun, how I would decide the issues presented.

The agency’s deviation from the law points to the second notable aspect of the bevacizumab story and the fundamental problem underlying all accelerated approvals. As an official noted,

[E]ven if you get the study, and you often do, sometimes they don’t confirm the efficacy of the product. […] What is FDA supposed to do with that? There’s now a huge and vocal constituency for the product. Whether or not the study showed it worked, there are people out there who think it worked, and lots of people with a financial stake in it. It becomes a political nightmare to try to take a product off the market that’s already developed that constituency by being approved for a period of time.[…][I]n some ways [it’s] a bigger problem than whether you get the data. It’s whether you can do anything with it when you have it.

This is precisely what happened with bevacizumab: the agency received over 400 submissions from patients, families, and others in the lead up to its public hearing.^35^ The Commissioner addressed patients directly and at considerable length in the body of her decision, seeking to assuage their concerns. Far from feeling the decision was disappointing but “reasonable,”^36^ many patients affected by the bevacizumab withdrawal expressed marked frustration, even anger, towards the agency.^37^

Relating the experience with bevacizumab back to the early days of HIV/AIDS and the pressure exerted by activists upon FDA, a long-time official expressed some optimism that the patients’ anger might transform with time:

[T]he HIV groups were very militant and everything in the early ‘90s and then they developed a relationship with the agency. And I think that that really made a difference in terms of how things went after that. They realized that the agency was not trying to obstruct, prevent them from getting treatment for their disease and everything. And also, they became more sophisticated about, we don’t want just any drug. At the beginning when there were no drugs, they really were willing to take anything. But then they realized that approving another drug that has perhaps greater side effects and maybe not as good a benefit isn’t enough. You know, it isn’t what we want. And so they really became more sophisticated in what they were asking for.

Yet there are signs that learning is occurring in both directions, that is, that patient advocacy may be reshaping agency thinking about when approval should be granted. In the case of aducanumab, for instance, Alzheimer’s disease patient groups convened a “listening session” with agency leadership after the November 2020 advisory committee meeting in which aducanumab’s efficacy was called into question. Afterwards Dr. Peter Stein, the head of FDA’s Office of New Drugs, reported that the agency had “heard very clearly from patients that they’re willing to accept some uncertainty to have access to a drug that could provide meaningful benefit in preventing the progression of [Alzheimer’s] disease”^38^ — a point that was echoed by the agency in the wake of aducanumab’s approval.

It is unlikely that the pressure exerted by patient advocacy organizations will abate as the agency has, through multiple pieces of legislation, been required by Congress to expand its patient engagement initiatives. However, there is no legislative direction about how specifically to engage patients in FDA decision-making or what weight patient perspectives should be granted relative to other considerations, such as the demonstrated efficacy of the drug.^39^

Following its experience with bevacizumab it appears that the agency prefers to provide sponsors with multiple opportunities to voluntarily withdraw an indication in the event that proves ineffective, pursuing withdrawal through a public hearing and the vitriol that it may spur in patient communities only as an option of last resort. Indeed, the ongoing case of “Makena” (hydroxyprogesterone caproate injection, a pre-term birth prevention drug originally granted accelerated approval in 2011, but which failed to confirm its efficacy post-approval) is proceeding far slower than bevacizumab.^40^ The hearing has yet to commence more than two years since agency reviewers recommended its withdrawal.^41^ And despite the express direction to the contrary in the regulations, separation of functions will once again be followed in an effort to give not only the sponsor, but also allied patients, an opportunity to be heard.^42^

### Eteplirsen: A More “Rational” System of Therapeutic Development

A third accelerated approval that holds important insights is eteplirsen (Exondys 51), a novel treatment for a subset of patients with Duchenne muscular dystrophy. Its approval in September 2016 followed months of discord both inside and outside the agency. The controversy stemmed from the limited evidence tendered by its sponsor, Sarepta, in support of its approval. The key study involved only 12 patients, with eight randomized to two different doses of eteplirsen and the remaining four patients given placebo for a period of 24 weeks. All twelve participants were then given eteplirsen and followed for a second period of 24 weeks. The key endpoint in the eteplirsen study was an increase in the presence of dystrophin in muscle biopsy specimens — a surrogate measure of the drug’s efficacy — that was measured at 12, 24, and 48 weeks of the study. In contrast, an earlier drug developed for the same disease had been tested in three randomized trials, comprising a total of 290 patients, which the FDA nevertheless rejected due to a lack of clear demonstrated clinical benefit as opposed to improvement in a surrogate marker.^43^ Eteplirsen was also associated with an improvement in a 6-minute walk test capacity of patients with Duchenne muscular dystrophy. But the association was shown in a post hoc calculation, which excluded 2 patients that had received eteplirsen during the study.^44^

FDA convened a public advisory committee meeting in April 2016 to review the data. Over a thousand people — many patients, families, and others all advocating passionately for the FDA to approve the drug notwithstanding the flaws in the trial evidence — attended. After hearing not only from patients and advocates, but also FDA staff and other experts, committee members arrived at a split vote about whether the agency should grant accelerated approval. Inside the agency, debate raged among scientific reviewers and agency leadership. All of the scientific reviewers responsible for evaluating the clinical studies, as well as key managers, including Dr. Ellis Unger (Director of the Office of Drug Evaluation I within the Center for Drug Evaluation and Research (CDER)), Dr. Luciana Borio (FDA’s acting Chief Scientist), and Dr. John Jenkins (Director of the Office of New Drugs) opposed approval. But Janet Woodcock, Director of CDER at the time, marshalled additional arguments in favour of giving eteplirsen the go ahead. In particular, she expressed concern that Sarepta “needed to be capitalized” and, failing approval of eteplirsen, the company itself would likely fold, pre-empting the possibility of ever determining whether the drug is clinically effective.^45^ The reviewers and managers appealed her decision, specifically citing her unusual involvement in the review process and repeated engagement with patient groups during the same period, before then-FDA Commissioner Dr. Robert Califf. However, Califf ultimately declined to interfere with the Woodcock’s decision.^46^

On the condition that a post-approval trial would be carried out to determine the effectiveness of eteplirsen by May 2021, the agency granted accelerated approval to Sarepta. However, before eteplirsen’s benefit was confirmed Sarepta was able to secure two subsequent accelerated approvals for two different sub-populations of Duchenne patients, each of which used the same exon skipping genomic technology and relied upon the same surrogate endpoint (i.e., dystrophin) to garner FDA’s market authorization. It appeared at one point that FDA might hold Sarepta to a higher standard but by late 2019 the agency capitulated, granting approval to the second Duchenne muscular dystrophy targeting drug, golodirsen (Vyondys 53).^47^ A third therapeutic (casimersen (Amondys 45)) followed in early 2021,^48^ illustrating how sponsors can expand their market size and reap increasing financial rewards^49^ even as the efficacy of the first accelerated approval remains uncertain.

Reflecting on the controversy circling around the eteplirsen decision, an agency official in a leadership role challenged the idea that FDA’s role is confined to reviewing and approving new therapies:

I think people tend to forget that the mandate of the FDA is not to approve or disapprove drugs, the mandate of the FDA is to public health. It is a science agency that has a regulatory function, and the regulatory function includes the use of drugs, biologics, and devices throughout their entire lifecycle. So, it’s always been important. But what’s changing is the information environment that we live in, and the possibility to come closer to what any, you know I would argue any knowledgeable person who’s lived in either the clinical world or the scientific development world for medical products, which is an iterative learning to diminish the uncertainty over time, not this threshold thing where you have one shot at getting on the market and then it’s bar the door whatever happens after that. *So, I think what you’re seeing is an effort by the FDA in conjunction with patient groups, largely, to come up with a more rational scheme for development and use of medical products.* (emphasis added)

In view of this broader scientific function, the official emphasized the importance of the agency adapting to this new “information environment,” where more can be learned about a drug’s safety and effectiveness after it enters into clinical use.

The same official was asked about whether there was room for improvement, for instance, in terms of the quality of post-approval studies. Research has shown that a significant percentage of drugs granted accelerated approval on the basis of a surrogate endpoint have been allowed to use the *same* surrogate endpoint in the post-approval study — a puzzling outcome for FDA to sanction given that post-approval studies are, by definition, intended to confirm clinical benefit as opposed to rely on a surrogate marker of the same.^50^ Still, the official was skeptical that using its authority to compel stronger study designs was the optimal way to proceed:

There’s a lot of room for improvement […] but I don’t think authority is the right answer to this, I really don’t. This is a clinical evidence issue that’s very complicated and involved in the ecosystem that needs a lot of work. But, having the FDA tell companies to do studies that don’t take into account that the use of the patients and families who are effected or the doctors and nurses and health systems that need to use the treatment […] I think it’s like a 1950’s concept. […] Some people just never got over that era where the FDA was regarded as some authority, doctors are stupid, patients don’t know any better, it’s a very authoritarian view of how the world ought to work. And it won’t be tolerated, at least in the US.

The official pointed instead to the need to redesign the entire system of evidence generation. Historically, “[w]e had to develop a clinical evaluation system that was pretty bizarrely separated from clinical practice” due to the fact that “healthcare records were kept on sheets of paper so there was no way to make sense of them.” In short, “we created a parallel universe of data and a whole set of rules to deal with that and to regulate the clinical trial space.” The result is that “we have a lot of focus on validity, but almost no focus on generalizability prior to approval.” Consistent with the agency’s growing appetite for “real world evidence,”^51^ the official envisioned an agency capable of harnessing a wider array of data, coupled with patient input throughout the drug development process, as essential elements of a more “rational” regulatory system.

## Regulatory Capture and Regulatory Modernization: Accelerated Approvals in Context

Aducanumab’s approval revived questions of “regulatory capture” that have long dogged the agency. The plan devised by agency officials seemingly in collaboration with representatives of Biogen, aducanumab’s sponsor, to secure the approval on the basis of a surrogate endpoint (i.e., the reduction of amyloid plaques) notwithstanding statements to the contrary made to an FDA advisory committee fits the capture narrative.^52^ However, the foregoing cases and interview data nuance this understanding of the agency in important ways.

First, regulatory capture is produced on several levels yet also offers an incomplete picture of the dynamic between the agency and the biopharmaceutical industry. The “revolving door” between FDA and industry is well documented: many FDA officials have departed for industry only to return a few years later (or vice versa), at times, trading on their experience in both environments to secure lucrative positions within consulting firms that advise sponsors on how to efficiently navigate the regulatory process.^53^ In some instances, these close-knit personal relationships translate into drug approvals despite minimal evidence of effectiveness (e.g., aducanumab) or engender delays to withdrawing a drug from the market (e.g., rofecoxib (Vioxx)) — each risking significant harm to patients.^54^ But regulatory capture also has structural underpinnings. Introduced in 1992 and renewed every five years since then, user fee legislation, which ties agency drug reviews to particular timelines in return for set fees from sponsors, has recast industry from a stakeholder in the system to one of the agency’s primary clients.^55^ As exemplified in the cases of gemtuzumab and eteplirsen, this client-focused approach filters through FDA’s work, prioritizing the next notable file for review over finalizing the design of an accelerated approval drug’s confirmatory trial. Whether at the inter-personal or structural level, though, the agency appears comfortable in its capture. At bottom, it guarantees continuity or a kind of “institutional incumbency” whereby the agency and industry remain the principal actors with an accepted division of labor.^56^ Sponsors are responsibilized with the task of generating the evidence in both the pre- and post-approval setting while FDA cooperatively and iteratively offers its feedback, as the evidence emerges.

Aducanumab’s approval revived questions of “regulatory capture” that have long dogged the agency. The plan devised by agency officials seemingly in collaboration with representatives of Biogen, aducanumab’s sponsor, to secure the approval on the basis of a surrogate endpoint (i.e., the reduction of amyloid plaques) notwithstanding statements to the contrary made to an FDA advisory committee fits the capture narrative. However, the foregoing cases and interview data nuance this understanding of the agency in important ways.

Second, while the agency as a whole is invested in preserving the status quo of *who* produces and regulates biopharmaceutical interventions, there is significant division within FDA’s ranks regarding *what* kinds of evidence are acceptable, especially at the point of market authorization. There is an epistemic divide within the agency between those who worry that the bar for approval has lowered significantly in recent years, and those who see rigid preferences for randomized controlled trials (RCTs) as missed opportunities to address unmet needs and better understand the generalizability of findings about new therapies with the improved collection of real world evidence (RWE). Research has shown that RWE is no substitute for an RCT.^57^ But an increasing percentage of FDA review decisions take observational studies and other RWE into account when approving a new drug.^58^ Further, Congress, with the support of the current FDA Commissioner, is poised to increase the agency’s mandate to incorporate RWE moving forward.^59^ Accelerated approvals are one notable — but far from the only — regulatory pathway where there has been a concerted move away from a rigid application of evidentiary standards in favor of a more flexible, discretion-driven approach that leans on a product’s full “lifecycle” to secure evidence and ensure that therapies like eteplirsen can reach patients before their sponsors fail financially. A number of internal critics of these and other similar decisions have left the agency^60^ and many scientific staff that are more accustomed to evaluating RCTs than RWE are approaching retirement. Referring to it during interviews as “CDER’s cliff,” long-tenured officials suggested a major transition within the agency’s personnel — its scientific culture and epistemological preference for data generated through RCTs — was afoot. In 2023 alone, FDA aims to hire over 200 new scientific staff in its drug review centers.^61^ Meanwhile, FDA officials that have embraced RWE hold positions of power, including the current Commissioner, Dr. Robert Califf, who championed RWE during his first term.^62^ The agency continues to explore, through new pilot pathways, opportunities beyond accelerated approvals to streamline drug development and regulatory decision-making.^63^

Third, regulatory capture — as a frame — underestimates the political acumen of the agency. The FDA has long been responsive to and, in more recent years increasingly adept at manoeuvring within, the wider political economy in which it operates. Without direction from Congress, it crafted the accelerated approval pathway on the fly under incredible pressure from HIV/AIDS patients and activists.^64^ And in the face of express legislative direction not to deploy its elaborate separation of functions procedure in the context of contemplating withdrawal of an accelerated approval indication, it chose to do precisely that in an effort to blunt pushback from the sponsor, patients, and allied opposition to FDA’s planned withdrawal of bevacizumab. This politicization of the agency has not happened overnight. The FDA has endured erosion of its legal authorities in the courts, whether to preclude the sale of experimental drugs to terminally ill patients,^65^ or to police off-label marketing by sponsors,^66^ and repeated challenges to its mandate from Congress, the Administration, and influential private actors, such as the Goldwater Institute and Manhattan Institute, in advancing populist ideas like the “right to try” that call into question the very existence of the agency.^67^ The shift in favor of expedited drug approval programs, RWE, and the inclusion of patients into its decision-making processes is not simply a response to changes in the law enacted by Congress. Rather, it has been informed by a calculated sense, as one official put it, of what will and will not be “tolerated” in the US. The agency is aware that integrating RWE and patient preferences into its decision-making is more of an art than a science at this stage.^68^ One interviewed official explained:

[T]here are a lot of attempts now to be more systematic about identifying specifically what kinds of diseases, what kinds of unmet needs warrant what level of uncertainty in approval and trying to make that more consistent across the reviewing divisions. That is incredibly hard both from a cultural perspective and just a logical perspective. How do you compare Alzheimer’s, to ALS, to breast cancer, to pancreatic cancer, and on and on? It’s not easy to quantify any of that.

“[D]ifferent medical reviewing divisions in FDA have different tolerances for uncertainty,” the official continued, thus the shift toward including patient preferences in the agency’s decision-making processes is fraught with institutional challenges.^69^ But the shift is part and parcel of a larger modernization strategy consciously crafted by the agency, diversifying the kinds of data that the agency relies upon, and approaching — with greater elasticity — the point in time at which it demands data about the safety and efficacy of a new therapy.^70^ As one official intimated, “there’s clearly a move towards using post-market […] to augment the fact that you have less data, or you’re going to use innovative data, even more innovative than what’s been used in the past, to drive the initial approvals.” The move is not extrinsic to FDA. Rather, it is designed in significant part by FDA, in concert with sponsors and other powerful actors seeking to maintain control over biopharmaceutical governance. When confronted with controversy, FDA seeks to preserve its discretion, working to resolve challenges in-house and forestall more radical change.^71^

## A Critical Appraisal of Reforms to the Accelerated Approval Pathway

Multiple bills were introduced in Congress during 2021-2022 with a view to altering — or supplanting—the accelerated approval pathway through which aducanumab entered the market. At one end of the spectrum is the *Promising Pathway Act,* which has been introduced in Congress on several occasions.^72^ It would create a new pathway that would grant “provisional approval” to therapies targeting life-threatening diseases, provided the sponsor supplies substantial evidence of safety and “relevant early evidence of positive therapeutic outcomes.”^73^ Positioning the bill as an answer to what its drafters perceive as the increasing demands of FDA, the *Promising Pathway Act* would further lower the bar for approval and sanction reliance upon RWE for confirmation of benefit.^74^ At the other end of the spectrum lies the *Accelerated Approval Integrity Act.* ^75^ When originally introduced, it envisioned several reforms to the accelerated approval pathway in view of the delays and enforcement challenges that have been observed, including measures that require post-approval trials to be underway at the point of approval, and automatic withdrawal of an accelerated approval five years after it enters the market if efficacy is not confirmed.

A third set of changes — occupying a middle ground between the lax *Promising Pathway Act* and stringent *Accelerated Approval Integrity Act* — were attached to the user fee legislative package developed in the House of Representatives.^76^ Initially, these changes appeared to have more political support.^77^ They, too, were ultimately dropped from the user fee legislation before it was passed hastily by Congress in order to avoid an FDA shutdown.^78^ However, these reforms recently returned (in slightly modified form) as part of the “omnibus” spending bill that was passed by Congress in late December 2022.^79^

Below, I apply the foregoing characterization of the FDA to three crucial elements of these newly enacted reforms. These three key elements map onto the challenges magnified by the cases of gemtuzumab, bevacizumab, and eteplirsen; namely, 1) the design and timely conduct of post-approval studies in respect of drugs granted accelerated approval; 2) the challenges associated with withdrawing an accelerated approval indication; and, 3) the agency’s stated move toward a more “rational,” flexible, and RWE-friendly regulatory system.

### Post-Approval Study Requirements, Timeliness, and Rigor

The first notable element concerns the timelines surrounding the design of post-approval studies in respect of an accelerated approval. The omnibus spending bill provides that the Secretary “shall specify the conditions for a post-approval study or studies required to be conducted…which may include enrolment targets, the study protocol, and milestones, including the target date of study completion.”^80^ Critically, this new mandatory requirement must also be satisfied “[n]ot later than the date of approval of a product under accelerated approval.”^81^ If consistently followed, this would mark a clear improvement over existing practice. At the same time, another provision in the same bill relays that post-approval studies may not always be required to confirm clinical benefit; in the event where no study is required by the agency, the Secretary must “publish…the rationale for why such study is not appropriate or necessary.”^82^

Apart from requiring that the “conditions” of the post-approval study be defined at the point of approval, this set of reforms largely preserves — if not expands — the agency’s discretion. Under existing regulations, post-approval studies are not always required. FDA officials claim that they have not, and never will, take advantage of that flexibility. But elevating that flexibility to the level of legislation adds legitimacy to the idea that clinical benefit need not be confirmed in certain circumstances even though accelerated approvals turn, during the premarket phase, on surrogate markers of efficacy.

History underscores this risk. Dating back to 1970, FDA’s regulations contemplated the use of studies relying on “historical controls” to support a new drug approval.^83^ However, the agency seldom cited studies using historical control data as part and parcel of a positive regulatory decision until recent years. For example, one study examining a twenty-year period (2000-2019) found that the agency relied on such data in 45 cases.^84^ Notably, thirty-three (or 73%) of those cases occurred during the latter ten years of that timeframe when FDA issued multiple guidances on the topic^85^ and Congress enacted the *21st Century Cures Act*,^86^ which sanctioned the use of RWE in regulatory decision-making.^87^ Sending the signal within the body of the legislation that the agency may, in circumstances left undefined in the bill, “not require that the sponsor…conduct a postapproval study” has the potential to soften the agency’s practice of demanding confirmatory studies across the board.

While the endpoints to be used for post-approval studies (if required) should be specified at the point of market entry, the omnibus bill is silent with respect to *what type* of endpoints — clinical versus surrogate — are to be relied upon to establish a therapy’s clinical benefit. This preserves FDA’s discretion and preferred division of labor: Agency officials can work out the finer details of post-approval studies on a case-by-case basis with sponsors.

### Withdrawal Challenges in the Face of Sponsor and Patient Opposition

A second key element of the recent reforms focuses on the problems involved in withdrawing an accelerated approval in the event that the sponsor fails to conduct the post-approval study in a timely manner, or does so, but the drug’s therapeutic benefits are not confirmed. Specifically, pursuant to the new legislation, withdrawals are to follow “expedited procedures” whereby the sponsor is provided with: i) “due notice;” ii) “an explanation for the proposed withdrawal;” iii) “an opportunity for a meeting with the Commissioner of Food and Drugs or the Commissioner’s designee;” and, iv) “an opportunity for a written appeal” to either the Commissioner or the Commissioner’s designee (“who has not participated in the proposed withdrawal of approval”).^88^ The new measure also allows for public comment on the proposed withdrawal and “convening and consulting an advisory committee on issues related to the proposed withdrawal, if requested by the sponsor.”^89^ In contrast to the current accelerated approval regulations, these reforms stop short of entitling sponsors to an oral hearing. Previously, sponsors were able to request a hearing within 15 days of receiving notice from the agency of the proposed withdrawal.^90^ That is no longer the case as a result of the new legislation.

On paper, these provisions promise to further expedite withdrawals when warranted relative to the current regulations. However, as cases like bevacizumab have shown, FDA may deviate from the letter of the law when under pressure from the sponsor and allied patient representatives. It is therefore foreseeable that if sponsors avail of an advisory committee under this new framework that the agency would, consistent with its current practices, use that as a public forum for hearing and trying to attend to patient interests. The new law’s allowance for an advisory committee to be convened may, in other words, amount to a public hearing wherein the agency is likely to struggle to balance meaningful and inclusive participation from those impacted by its decision^91^ with its core function of ensuring the safety and efficacy of drugs.^92^

A more optimal way to proceed given the vexed nature of withdrawals would be to take the decision out of FDA’s hands altogether. In other jurisdictions, this is how several similarly expedited approval pathways work: if a post-approval study is not completed within a set timeframe or fails to demonstrate the clinical benefit of the therapy, then the indication is automatically withdrawn. As FDA officials have been at pains to point out, that is not true of accelerated approvals.^93^ The *Accelerated Approval Integrity Act* provided for automatic withdrawal.^94^ However, the measure of automatic withdrawal was not carried through the two proposed pieces of user fee legislation that the agency helped craft, nor the omnibus spending bill that has now become law. Under this discretion-based approach, decisions to potentially withdraw an accelerated approval are likely to continue to be highly politicized.

### Increasing Evidentiary Flexibility Moving Forward

The final element of the new reforms that merits attention is about what kinds of evidence will suffice, either to garner an accelerated approval, or to confirm its benefit post-approval. The amendments to the accelerated approval pathway that were proposed during the user fee legislation negotiations included a provision that, on the one hand, reinforced the agency’s authority to compel a sponsor to conduct one or more post-approval studies while, on the other hand, noted that such studies “may be augmented or supported by real world evidence” (RWE).^95^ This explicit mention of RWE was removed from the omnibus bill that Congress passed in late 2022. However, the legislation calls upon FDA to develop and issue various new guidance, including about: “how sponsor questions related to the identification of novel surrogate or intermediate clinical endpoints may be addressed in early-stage development meetings” with the agency; “considerations related to the use of surrogate or intermediate clinical endpoints that may support the accelerated approval of an application”; and, “the use of novel clinical trial designs that may be used to conduct appropriate post-approval studies.”^96^ It appears, then, that FDA has ample discretion to describe how “novel” types of studies, including, RWE might support or confirm an accelerated approval.^97^

On its face, this added source of discretion and flexible approach to evidence generation is not alarming. It is in line with current research which indicates that RWE can complement — but not replace — the evidence generated through RCTs.^98^ However, legitimizing the agency’s flexible evidentiary standards should be interpreted in view of the agency’s track record with accelerated approvals to date. The FDA has been the subject of significant critique for allowing sponsors not only to use a surrogate endpoint to secure an accelerated approval (which has always been sanctioned under that pathway), but also in the course of a post-approval study to confirm a therapy’s clinical benefit. That pattern played out in over 40% of oncology related accelerated approvals granted between 1992 and 2017.^99^ Silent on the issue of what endpoints FDA should rely upon while also empowering the agency to develop new guidances surrounding “surrogate and intermediate clinical endpoints” as well as the design of novel post-approval studies, and the omnibus spending bill may perpetuate the problem of weak post-approval studies provided that supportive RWE and/or other data is also supplied by the sponsor. For those who are concerned that the regulator’s approval standards have, under the agency’s watch, already diminished too far,^100^ this kind of change has little to offer in terms of restoring the agency’s commitment to public health through the encouragement of strong, scientific evidence production.

## The Case for More Structural Reform

In the wake of aducanumab FDA has taken action to redress several “dangling” accelerated approvals that have not confirmed clinical benefit after years on the market,^101^ signalling that post-approval trials must not only be fully designed but underway for a therapy to receive accelerated approval while welcoming legislative reforms to the accelerated approval pathway. Several reforms have since come to pass as part of the omnibus spending legislation, however, none of them stand to limit the agency’s discretion. The new law does not stipulate the types of endpoints that are acceptable for the purposes of a post-approval study, nor dictate when to withdraw an accelerated approval. Rather, the agency has proactively molded these reforms in line with its own institutional priorities.

Arming the agency with the power to articulate its evidentiary expectations for accelerated approvals through new guidances, the omnibus spending bill ensures that any further trouble-shooting of the pathway will occur in-house. Specifically, the bill directed FDA to “establish an intra-agency coordinating council…to ensure the consistent and appropriate use of accelerated approval across the Food and Drug Administration.”^102^ Composed exclusively of senior officials, including the directors of CDER, CBER, the Oncology Center of Excellence, the Office of New Drugs, the Office of Orphan Products Development, the Office of Tissues and Advanced Therapies (OTAT), and at least three directors of review divisions that oversee accelerated approvals, such as the Office of Neuroscience, the new council must work with product review teams to “support the consistent and appropriate use of accelerated approvals” and report annually on its progress.^103^ Notably, all of the senior officials named to the council have figured prominently in controversial accelerated approvals in recent years, including Dr. Billy Dunn, the Director of the Office of Neuroscience, which approved aducanumab, or oversee agency divisions that are expected to become key growth areas, especially OTAT given the emergence of gene therapies incorporating CRISPR-technology. FDA is — as emphasized by several interviewed officials — keenly aware of the challenges involved in achieving “consistency” across therapeutic classes because different reviewing divisions have “different tolerances for uncertainty.” Yet, the agency asked for a council to smooth these differences within FDA and Congress answered in the affirmative, fundamentally preserving the agency’s control over its own regulatory processes.

In the wake of aducanumab FDA has taken action to redress several “dangling” accelerated approvals that have not confirmed clinical benefit after years on the market, signalling that post-approval trials must not only be fully designed but underway for a therapy to receive accelerated approval while welcoming legislative reforms to the accelerated approval pathway. Several reforms have since come to pass as part of the omnibus spending legislation, however, none of them stand to limit the agency’s discretion.

Meanwhile, with all the attention on aducanumab, the agency has opened up alternative pathways that import some — but not all — of the features of accelerated approval. Announced as part of its commitments under the user fee legislation, the “Split Real Time Application Review” (STAR)^104^ pilot program reduces review times (from the six months period that normally applies to priority reviews down to five months) and allows sponsors to rely upon surrogate markers *without* a requirement to conduct confirmatory trials following market entry.^105^ Combined with the reforms to accelerated approvals that have been adopted, the STAR program is indicative of the agency’s core priorities of preserving an established division of labor with sponsors, giving precedence to new therapies over post-approval studies in its workflow, and maintaining — at all cost — its discretion to make decisions, in line with its strategic goals of improving patient engagement and expanding the types of evidence it incorporates into its new drug reviews, on a case-by-case basis. The problem, in other words, runs deeper than aducanumab; it rests with the agency and its decision, abetted by Congress, to bend its evidentiary standards in favor of flexibility.

Altering this state of affairs requires structural reform. Below I outline two reforms that aim to restructure *how* the agency allocates its resources and who renders decisions in response to post-approval study findings. The first reform is tied to user fee legislation, specifically, recalibrating the ways in which that legislation structures and drives the work of the agency. The second reform, inspired by the agency’s “Drug Efficacy Study Initiative” (DESI) of the 1960s,^106^ seeks to disturb the control currently shared by the agency and sponsors over the biopharmaceutical knowledge production.

### Recalibrating the Priorities Behind User Fee Legislation

Over the course of six consecutive user fee legislative packages dating back to 1992, FDA has hastened its drug review times and reduced the number of review cycles needed for a new molecular entity to receive approval.^107^ This has engendered a more cooperative relationship between agency and industry.^108^ Absent the political will to fund FDA operations entirely via the public purse, user fee legislation constitutes one means to re-align FDA’s priorities.

Before user fee bills are drafted, FDA prepares a “commitment letter,” outlining various priorities that it plans to achieve over the course of the term of the next user fee legislative package in exchange for user fees from industry. In addition to creating the STAR pilot program noted above, the most recent commitment letter particularized several priorities, including creating new types of meetings for sponsors to “achieve earlier and more interactive communication with FDA staff;” hiring over 200 new staff in its product review centers; moving “more resources and activities to cloud-based platforms and increase use of digital health technology-generated data;” and “incorporating more real-world evidence into…[the] analytic strategies” used in FDA’s “Sentinel initiative,” the agency’s post-approval safety detection system — all of which were lauded by industry.^109^ FDA also plans to initiate a new “Advancing RWE program” that “seeks to improve the quality and acceptability of RWE-based approaches in support of new intended labeling claims, including approval of new indications of approved medical products or to satisfy post-approval study requirements.”^110^

The commitment letter does contain some positive statements from the point of view of positioning post-approval studies to be underway when new therapies enter the market. Specifically, the letter states that the agency will “communicate details on anticipated” post-approval study requirements no later than 8 or 6 weeks prior to the target approval date for standard and priority reviews, respectively.^111^ But the letter is silent with respect to how user fee resources will be allocated to improve the enforcement of post-approval study requirements and agency decision-making if and when they are fulfilled. In short, the proposed user fee legislation, and FDA’s commitment letter underpinning it, largely preserves the agency’s forward-looking focus rather that prioritizing and redistributing resources to the post-approval phase of a product’s lifecycle. This is why the agency’s claim of adopting a lifecycle approach to regulation rings hollow. On the one hand, its reviewing divisions are, under the accelerated approval pathway and other expedited programs, leaning more and more on the post-approval phase of a drug to learn about its safety and effectiveness. On the other, the resources to support that institutional shift — to ensure that post-approval studies are designed prior to product launch, and to make sense of, and act upon, the study findings as they roll in — have not followed.

To break this pattern, user fee legislation and the agency’s commitment letter should be modified in two ways. First, the legislation should stipulate that resources gained from user fees should be re-allocated to units within FDA that have the expertise to act upon evidence generated post-approval. At present, power within the FDA continues to reside primarily within its reviewing divisions. Under the rubric of lifecycle regulation, clinician reviewers today work closely with the agency’s epidemiologists and other methodologists who are better equipped to assess safety and effectiveness in a noisy post-approval environment. Yet, decision-making authority regarding whether to issue safety warnings, alter a novel therapeutic’s labeling, or withdraw an indication from the market is vested exclusively in the reviewer side of the house.^112^ If FDA is genuinely committed to incorporating more RWE into its regulatory process, it must mitigate the epistemic divide between those who serve as gatekeepers to the market (e.g., officials within the Office of New Drugs), and those charged with monitoring drug safety and efficacy in the real world (i.e., staff within the Office of Surveillance and Epidemiology). If the intention is to put more emphasis on RWE it is imperative that those best positioned to assess the strength of that knowledge enjoy a corresponding level of resources and decision-making power.

Second, the legislation’s model of tying review time targets to user fees should be qualified by sponsors’ post-approval study preparatory performance. FDA currently aims to complete reviews of “priority” products, including accelerated approval applications, within six months of the agency’s determination that the sponsor’s submission warrants a full review.^113^ Anticipating that post-approval study designs should be in place by the point of approval, the agency’s current commitment letter also contemplates a meeting between staff and the sponsor about post-approval study designs no later than six weeks prior to that six-month review deadline. To underscore the importance of that task, however, the agency’s commitment letter should specify that the six-month review time does *not* apply to accelerated approval applications that lack clearly defined post-approval study protocols by the time of the meeting between agency staff and the sponsor.

### Disrupting Incumbents’ Control Over Biopharmaceutical Knowledge

The cases of aducanumab, gemtuzumab, bevacizumab, and etiplersen call into question the entire premise of lifecycle regulation; namely, that the agency can and will act as new information about a therapy’s safety and effectiveness develops. As this analysis has shown, the agency is a political actor, with defined priorities, which precipitate decisions such as aducanumab. To tackle this fundamental problem, one approach is to change who is responsible for deciding when to act. In this regard, the FDA’s own history is instructive.

When the power to require substantial evidence of effectiveness was formally added to FDA’s mandate in 1962, the agency decided to assess not just all new drugs approved from then on against that standard, but also all new drugs approved between 1938 and 1962 (the period in which a demonstration of safety alone was formally required for market approval). This presented an immediate resource issue: thousands of old, potentially ineffective, drugs had entered the market during that period. To assist in the evaluation of these old drugs, the FDA enlisted the National Academy of Sciences (NAS) and created the program of “Drug Efficacy Study Initiative,” which by the time it concluded its work in the early 1980s, had assessed the efficacy of more than 3,400 old drugs.^114^ Under the auspices of the NAS, 180 specialist physicians and researchers assembled into 30 panels corresponding to the main types of drugs under review, evaluated efficacy using briefs submitted by the sponsor, files from FDA and relevant medical literature identified by the specialists. Each panel delivered its recommendation to a Policy Advisory Committee and in turn FDA for final decision-making.

Sponsors challenged the FDA’s DESI program in Court. However, the US Supreme Court upheld the FDA’s authority to create such an expedited procedure for removing products from the market.^115^ And while subsequent changes in the law may no longer render such procedures immune from judicial review,^116^ the agency has continuing authority to craft and implement summary-type procedures to assess the safety and effectiveness of a drug based on the available evidence.^117^

More importantly, the DESI program stands as an important example of FDA working in collaboration with outside actors to assess drug safety and effectiveness. If the FDA is unlikely to require rigorous post-approval study designs to confirm the efficacy of accelerated approvals^118^ or, in the wake of cases like bevacizumab, unable to swiftly to act when post-approval studies fail to confirm a drug’s clinical benefit, then Congress should convene a new body, with suitable scientific expertise and independence from FDA, industry, and patients, to assist in its decision-making. Designing such a body and ensuring it has sway over regulatory decision-making is not straightforward: three experts resigned from the agency’s advisory committee after FDA approved aducanumab.^119^ However, if the aim of reform is to renew the regulatory system’s core function of producing valuable scientific knowledge about the safety and effectiveness of new therapies, outside assistance may be essential. There is no indication that FDA’s embrace of its expedited programs, including accelerated approvals, or that its efforts to accommodate more diverse forms of evidence such as RWE, will otherwise abate.

## Conclusion

Shifts in FDA law and policy are often tethered to a controversial regulatory decision. Granting accelerated approval to aducanumab for the treatment of Alzheimer’s disease on the basis of minimal evidence that it worked prompted investigation and ultimately legislative reform. While the recent changes to the accelerated approval appear helpful on balance, a deeper understanding of the agency — of its institutional priorities, how it allocates resources within its ranks, and the larger political economy in which it operates — suggests that more structural changes are needed to restore FDA’s fundamental mission of securing the production of high-quality information about the safety and effectiveness of new therapies and acting upon that information as it evolves.

## Note

Matthew Herder is a member of the Patented Medicine Prices Review Board (PMPRB), Canada’s national drug pricing regulator, and receives honoraria for his public service. The PMPRB had no role whatsoever in the design or conduct of the research, or the analysis and writing of this manuscript. Research for this article was supported by a grant from the Canadian Institutes of Health Research (CIHR PJT 156256) and an award from the Commonwealth Fund.
